# Feasibility of Using Text Messaging to Identify and Assist Patients With Hypertension With Health-Related Social Needs: Cross-Sectional Study

**DOI:** 10.2196/54530

**Published:** 2024-02-13

**Authors:** Aryn Kormanis, Selina Quinones, Corey Obermiller, Nancy Denizard-Thompson, Deepak Palakshappa

**Affiliations:** 1 Department of Internal Medicine Wake Forest University School of Medicine Winston Salem, NC United States; 2 Department of Pediatrics Wake Forest University School of Medicine Winston Salem, NC United States; 3 Department of Epidemiology and Prevention Wake Forest University School of Medicine Winston Salem, NC United States

**Keywords:** social determinants of health, health-related social needs, mobile health, health information technology, feasibility, mobile phone, SMS text messaging, message, pilot study, patients, patient, hypertension, screening

## Abstract

**Background:**

Health-related social needs are associated with poor health outcomes, increased acute health care use, and impaired chronic disease management. Given these negative outcomes, an increasing number of national health care organizations have recommended that the health system screen and address unmet health-related social needs as a routine part of clinical care, but there are limited data on how to implement social needs screening in clinical settings to improve the management of chronic diseases such as hypertension. SMS text messaging could be an effective and efficient approach to screen patients; however, there are limited data on the feasibility of using it.

**Objective:**

We conducted a cross-sectional study of patients with hypertension to determine the feasibility of using SMS text messaging to screen patients for unmet health-related social needs.

**Methods:**

We randomly selected 200 patients (≥18 years) from 1 academic health system. Patients were included if they were seen at one of 17 primary care clinics that were part of the academic health system and located in Forsyth County, North Carolina. We limited the sample to patients seen in one of these clinics to provide tailored information about local community-based resources. To ensure that the participants were still patients within the clinic, we only included those who had a visit in the previous 3 months. The SMS text message included a link to 6 questions regarding food, housing, and transportation. Patients who screened positive and were interested received a subsequent message with information about local resources. We assessed the proportion of patients who completed the questions. We also evaluated for the differences in the demographics between patients who completed the questions and those who did not using bivariate analyses.

**Results:**

Of the 200 patients, the majority were female (n=109, 54.5%), non-Hispanic White (n=114, 57.0%), and received commercial insurance (n=105, 52.5%). There were no significant differences in demographics between the 4446 patients who were eligible and the 200 randomly selected patients. Of the 200 patients included, the SMS text message was unable to be delivered to 9 (4.5%) patients and 17 (8.5%) completed the social needs questionnaire. We did not observe a significant difference in the demographic characteristics of patients who did versus did not complete the questionnaire. Of the 17, a total of 5 (29.4%) reported at least 1 unmet need, but only 2 chose to receive resource information.

**Conclusions:**

We found that only 8.5% (n=17) of patients completed a SMS text message–based health-related social needs questionnaire. SMS text messaging may not be feasible as a single modality to screen patients in this population. Future research should evaluate if SMS text message–based social needs screening is feasible in other populations or effective when paired with other screening modalities.

## Introduction

Unmet health-related social needs, such as food insecurity and housing instability, are associated with impaired chronic disease management and worse health outcomes [1-5]. For example, people with hypertension who live in a food-insecure household are more likely to have worse diet quality and blood pressure control than people with hypertension who live in a food-secure household. Because of their negative impact, national organizations, such as the Centers for Medicare and Medicaid (CMS), have recommended that health systems integrate interventions to screen and address patients’ unmet social needs as a routine part of clinical care [6-9]. Although there has been growing investment by health systems to integrate these interventions, there are still limited data on how to most effectively implement screening in busy clinical settings [10,11]. Studies assessing the use of mobile tools (eg, tablets) and telephone-based screening identified barriers to these screening modalities [12-14]. Barriers to using these methods include that tablets are dependent on patients presenting in person to the clinic [12,15,16] and phone-based screening may add additional work for already busy clinical staff [14].

SMS text messaging could be an effective and efficient approach to assess patients for health-related social needs and allow for screening to occur outside of the direct patient encounter. However, prior studies have not used SMS text messaging to screen patients for health-related social needs. To fill this gap, our objective was to determine the feasibility and acceptability of using SMS text messaging to screen and assist patients with hypertension with health-related social needs. We were specifically interested in evaluating if patients would complete a SMS text message–based social risk questionnaire and if there were differences in demographics between patients who completed the questionnaire and those who did not.

## Methods

### Study Design and Population

We conducted a pilot cross-sectional study at Atrium Health Wake Forest Baptist Health (AHWFB) to assess the feasibility of using SMS text messaging to send a link to a web-based questionnaire to screen patients for health-related social needs. AHWFB is a large, integrated academic health system serving communities in Central and Western North Carolina. The system is comprised of a tertiary care hospital located in Winston-Salem, NC, 4 community hospitals, and >300 ambulatory practices that all use a single electronic health record (EHR; EpicCare). We identified eligible adult patients (≥18 years) with hypertension who were seen at an AHWFB primary care clinic in the previous 3 months (between November 2022 and February 2023). We only included patients who had been seen in the last 3 months to ensure they were still a patient at the clinic. We limited the sample to patients seen in an AHWFB internal or family medicine clinic (17 clinics) in Forsyth County, North Carolina to provide tailored information about local community-based resources (eg, food pantries). We also included these 17 clinics because they are in the process of integrating social risk screening into routine clinical care, but none of the 17 clinics had implemented a standardized screening process prior to or during the time period the study was conducted. Based on 2023 census estimates of people living in Forsyth County, 17.2% of the population are older than 65 years of age, 65.7% identify as non-Hispanic White, 27.7% as non-Hispanic Black, and 14.3% as Hispanic. A total of 14% of the population have a household income below the federal poverty level, 12.9% of the population are estimated to be uninsured, and 11.6% of households in the county are estimated to be food insecure. We recruited participants by taking a simple random sample of 200 patients from the 4446 eligible patients identified and sending them SMS text messages to the cell phone number included in the EHR. As there is no specific consensus on the sample size necessary to assess the feasibility of a pilot study, consistent with prior studies, we included 200 (~5% of the eligible population) patients [17,18].

### Ethical Considerations

The Wake Forest University School of Medicine institutional review board reviewed and approved this study under the expedited review with a waiver of written informed consent (IRB00092658). The SMS text message included language to notify the participants that the questionnaire was part of a research study and that participation was voluntary. To maintain privacy and confidentiality, all participants’ responses to the social risk questionnaire and personal health and demographic data were stored in a password-protected file on the institution’s secure server. Only study team members had access to the data. The participants did not receive compensation for participation in the study.

### SMS Text Messages

We developed the SMS text message–based on a detailed literature review of the social needs literature (Multimedia Appendix 1). The AHWFB digital communication committee also reviewed the SMS text messages. The committee includes patients, clinicians, hospital administrators, and members of the institutional review board. The committee reviews all SMS text messages that may be sent to patients for either research or clinical purposes at the institution, and they provide input on the wording of the messages. We also reviewed the SMS text messages with patients who were not included in the study to assess face validity and to provide additional input on the messages. The SMS text message included a link to a questionnaire with 6 questions from the CMS Accountable Health Communities Health-Related Social Needs Screening Tool [7]. The 6 questions included 2 questions about housing (including 1 about patients’ current housing situation and 1 about problems with housing), 2 questions about food insecurity, 1 question about transportation, and 1 question about use. We limited to questions regarding food, housing, transportation, and utilities because there were local resources available to assist patients with these domains. The initial message was sent to the patient’s cell phone number listed in the EHR on March 14, 2023. All of the 4446 patients eligible had a phone number listed in the EHR. For those that did not respond, the same message was sent again 1 week later. Responses were documented in the EHR. The SMS text message and questionnaire were sent in English or Spanish based on the preferred language of the patient listed in the EHR. We used the standard scoring to identify patients who screened positive for a social need. The patients were identified as having housing instability if they provided a response other than “I have a steady place to live” to the first housing question or if they provided a response other than “None of the above” to the second question. Patients were identified as having food insecurity if they responded to either of the 2 questions with “sometimes true” or “often true.” Patients were identified as having a lack of transportation if they responded “yes” to the transportation question, and they were identified as having difficulty with utilities if they responded “yes” or “already shut off” to the utilities question.

For patients who screened positive for any of the social needs, they were asked if they would be interested in receiving information about local community resources. A list of resources was then sent by the system in a subsequent SMS text message to patients who were interested in receiving information about local community resources. The system used branching logic and only provided information on resources for the unmet need the patient reported (eg, food resources for those with food insecurity). Patients were asked if they would be willing to receive an additional message 1 month later to assess the acceptability of the process and if they used any of the information provided about community resources (Multimedia Appendix 1). Questions were based on the validated Acceptability of Intervention Measure [19].

### Statistical Analysis

We obtained age, sex, race (White, Black, American Indian or Alaska Native, Asian Indian, Filipino, and other), ethnicity (Hispanic and non-Hispanic), insurance status (commercial or private, Medicaid, Medicare, uninsured, and other), and preferred language (English or Spanish) for all patients through data extraction from the EHR. Informed by the Reach, Effectiveness, Acceptability, Implementation, and Maintenance (RE-AIM) framework [20-22], feasibility was based on the reach of the screening or the proportion of patients who completed the social needs questionnaire. To understand if using a SMS text message–based social needs questionnaire could lead to disparities in who completes screening questions, we evaluated for differences in demographics between patients who completed the questions and those who did not use bivariate analyses. We used either the chi-square or Fisher exact test for categorical variables, and we used the Welch *t* test for continuous variables to account for unequal sample sizes and variances. We considered an α of <.05 significant and all analyses were conducted using Stata 15.0 (StataCorp).

## Results

Of the 200 patients randomly selected, the majority were females (n=109, 54.5%), non-Hispanic White (n=114, 57.0%), who received commercial or private health insurance (n=105, 52.5%), and had English listed as their preferred language (n=192, 96.0%). The mean age of the patients selected was 57.6 (SD 12.9) years of age.

Of the 200 patients, the SMS text message was unable to be delivered to 9 patients (either because the number listed was no longer working or was not a cell phone). A total of 17 (8.5%) patients completed the social needs questionnaire (Table 1).

We did not find a significant difference in demographics between patients who did and those who did not complete the questionnaire. Of the 17 who completed the questionnaire, 5 (29.4%) reported at least 1 unmet social need, but only 2 chose to receive local resource information. One person completed the follow-up questionnaire and reported that they learned about new community resources through the process.

**Table 1:** Study patient characteristics^a^.

Characteristics			Total (N=200)	Did not respond (n=183)		Responded (n=17)		*P* value
Age (years), mean (SD)			57.6 (12.9)	57.1 (13.0)		63.1 (11.6)		.05
**Sex, n (%)**								.16
	Male	91 (45.5)		86 (47.0)	5 (29.4)			
	Female	109 (54.5)		97 (53.0)	12 (70.6)			
**Race, n (%)**								.99
	Non-Hispanic and White	114 (57.0)		104 (56.8)	10 (58.8)			
	Non-Hispanic and Black	67 (33.5)		61 (33.3)	6 (35.3)			
	American Indian or Alaska Native	1 (0.5)		1 (0.6)	0 (0.0)			
	Asian Indian	3 (1.5)		3 (1.6)	0 (0.0)			
	Filipino	1 (0.5)		1 (0.6)	0 (0.0)			
	Other	14 (7.0)		13 (7.1)	1 (5.9)			
**Ethnicity, n (%)**								.99
	Hispanic, Latino, or Spanish	15 (7.5)		14 (7.7)	1 (5.9)			
	Not Hispanic, Latino, or Spanish	185 (92.5)		169 (92.4)	16 (94.1)			
**Health insurance, n (%)**								.17
	Commercial	105 (52.5)		100 (54.6)	5 (29.4)			
	Medicaid	13 (6.5)		12 (6.6)	1 (5.9)			
	Medicare	64 (32.0)		55 (30.1)	9 (52.9)			
	Uninsured	11 (5.5)		10 (5.5)	1 (5.9)			
	Other	7 (3.5)		6 (3.3)	1 (5.9)			
**Language, n (%)**								.52
	English	192 (96.0)		176 (96.2)	16 (94.1)			
	Spanish	8 (4.0)		7 (3.8)	1 (5.9)			
**Food insecurity, n (%)**								
	Yes	N/A^b^		N/A	3 (17.7)		N/A	
	No	N/A		N/A	14 (82.4)		N/A	
**Living situation, n (%)**								
	I have a steady place to live	N/A		N/A	16 (94.1)		N/A	
	Prefer not answer	N/A		N/A	1 (5.9)		N/A	
**Problems in the home, n (%)**								
	None	N/A		N/A	16 (94.1)		N/A	
	Pests (eg, bugs, ants, or mice)	N/A		N/A	1 (5.9)		N/A	
**Lack of transportation, n (%)**								
	No	N/A		N/A	14 (87.5)		N/A	
	Yes	N/A		N/A	1 (6.3)		N/A	
	Prefer not answer	N/A		N/A	1 (6.3)		N/A	
**Electric, gas, or water shut off, n (%)**								
	No	N/A		N/A	17 (100.0)		N/A	

^a^Bivariate analysis comparing characteristics of patients who were did and did not respond to a SMS text message linked social needs questionnaire in March 2023; responses to the social needs questionnaire for the 17 participants are also included.

^b^N/A: not applicable.

## Discussion

These results suggest that SMS text messaging may be inadequate when used as a single modality for screening patients for unmet health-related social needs in this population, as only 17 (8.5%) of patients completed the social needs questionnaire. Yet 29% (n=5) of patients who completed the questionnaire reported having at least 1 unmet social need. Given the growing investment in integrating social care interventions into health care delivery, understanding screening strategies that are both effective and those that may be less effective are important.

There are several possible explanations for the low response rate. First, patients could have concerns about completing the social risk questionnaire using a SMS text message–based link. Prior studies have found that there are multiple factors, such as trust in their provider and concern about disclosure of sensitive information, contributing to patients’ acceptability of social needs screening [23-25]. Previous studies have also found that patients are more likely to complete social risk questionnaires and disclose sensitive information if they are screened using paper or tablets in the clinic, rather than being verbally asked [26-29]. Patients may be unable to have confidence in who is administering the questionnaire and have access to the results using SMS text messaging. We did not notify patients that they would be sent the SMS text message. Discussing with patients prior to sending the message or directly tying the message to an upcoming visit may result in higher screening completion rates. It is also possible that the majority of patients who received the message did not have a social need, so they did not see a benefit in completing the social risk questionnaire.

A second possibility for the low response rate is the wording of the message. We tried to gather input from multiple different stakeholders in developing the message, but patients may have either misunderstood the purpose of the SMS text message or were not interested in participating in a research study. A SMS text message coming directly from a patient’s provider or clinic could yield different results and future research could randomize who sends the message. A third reason for the low response rate could be barriers to using the technology [30,31]. We had to embed the questionnaire as a link in the SMS text message rather than have the questions directly in the message. Barriers to accessing the link could include patients not having a smartphone, although more than 90% of people in the United States have a smartphone [32]. Even if patients had a smartphone, patients may not have had access to the internet on the device to access the link. Another barrier to using the technology could have been that people were concerned about accessing a link on their phone, because of concerns about privacy, or were unsure of how to access the link.

Despite the low response rate and the limitations, this study provides important information for clinical care. This is the first study to assess patients’ health-related social needs using SMS text messaging, and the response rate was lower than what has been seen in other SMS text messaging–based patient-reported outcomes studies [33,34]. Numerous national health care organizations have recommended that health systems address patients’ unmet social needs as a routine part of clinical care, and CMS will require that all adult patients admitted to the hospital be screened for health-related social needs beginning in 2024 [6-9]. Many health systems are in the process of implementing different approaches to screen patients for social needs. At least in this population, simply sending out a SMS text message with the social risk questionnaire may not be effective as a single modality to assess all patients for health-related social needs. Health systems and clinics may need to implement multiple modalities, such as using SMS text messaging, the patient portal, or tablets in the clinic, to effectively screen all patients. If health systems are still interested in using SMS text messaging, they may also want to consider varying when and how the messages are sent.

There are several limitations to this study that should be acknowledged. First, although we included patients seen in 17 different primary care clinics, the clinics were all located in the same county and part of the same academic medical center so the results may not be generalizable to other populations. Second, all of the messages were sent at the same time and day for every patient. Varying when the message is sent (ie, time of the day and proximity to a clinic visit), may yield different results. Third, the message was sent based on the preferred language listed in the EHR. It is possible that the language listed was not correct. Fourth, we limited this study to patients with a diagnosis of hypertension. Future studies in other patient populations could find different results. Fifth, as in other studies screening patients for social risks in clinical care settings, individuals who have a primary care provider or clinic may be different than individuals who do not (ie, more likely to have health insurance) [35]. The results of the social risk questionnaire may not be representative of the surrounding community. Sixth, the SMS text message was unable to be delivered to 9 patients based on the phone number included in the EHR. As populations who have been socially and economically disadvantaged are more likely to have disruptions in their phone service, future research should evaluate the reasons why the message was unable to be delivered.

In this study, we found that only 17 (8.5%) of the 200 randomly selected patients completed a SMS text message–based health-related social needs questionnaire. Despite the negative results, this study provides important information for clinics considering implementing social needs screening. Further research is needed to understand how to most effectively and efficiently implement social needs screening in a patient-centered approach.

## Appendix

Multimedia Appendix 1 Data and questions included in text messages.

## Abbreviations

AHWFB: Atrium Health Wake Forest Baptist Health
CMS: Centers for Medicare and Medicaid
EHR: electronic health record
RE-AIM: Reach, Effectiveness, Acceptability, Implementation, and Maintenance

## Data Availability

The data sets generated and analyzed during this study are not publicly available because they contain personal health information, but deidentified data sets are available from the corresponding author on reasonable request.

## References

[1] Berkowitz SA, Basu S (2021). Unmet social needs and worse mental health after expiration of COVID-19 federal pandemic unemployment compensation. Health Aff (Millwood).

[2] Berkowitz SA, Palakshappa D, Seligman HK, Hanmer J (2022). Changes in food insecurity and changes in patient-reported outcomes: a nationally representative cohort study. J Gen Intern Med.

[3] Berkowitz SA, Seligman HK, Meigs JB, Basu S (2018). Food insecurity, healthcare utilization, and high cost: a longitudinal cohort study. Am J Manag Care.

[4] Seligman HK, Berkowitz SA (2019). Aligning programs and policies to support food security and public health goals in the United States. Annu Rev Public Health.

[5] Palakshappa D, Garg A, Peltz A, Wong CA, Cholera R, Berkowitz SA (2023). Food insecurity was associated with greater family health care expenditures in the US, 2016-17. Health Aff (Millwood).

[6] National Academies of Sciences, Engineering, and Medicine, Health and Medicine Division, Board on Health Care Services, Committee on Integrating Social Needs Care into the Delivery of Health Care to Improve the Nation's Health (2019). Integrating Social Care into the Delivery of Health Care: Moving Upstream to Improve the Nation's Health.

[7] (2018). Accountable health communities model. Centers for Medicare and Medicaid Services.

[8] Parish W, Beil H, He F, D'Arcangelo N, Romaire M, Rojas-Smith L, Haber SG (2023). Health care impacts of resource navigation for health-related social needs in the accountable health communities model. Health Aff (Millwood).

[9] Renaud J, McClellan SR, DePriest K, Witgert K, O'Connor S, Johnson KA, Barolin N, Gottlieb LM, De Marchis EH, Rojas-Smith L, Haber SG (2023). Addressing health-related social needs via community resources: lessons from accountable health communities. Health Aff (Millwood).

[10] Gottlieb L, Cottrell EK, Park B, Clark KD, Gold R, Fichtenberg C (2018). Advancing social prescribing with implementation science. J Am Board Fam Med.

[11] Gold R, Kaufmann J, Cottrell EK, Bunce A, Sheppler CR, Hoopes M, Krancari M, Gottlieb LM, Bowen M, Bava J, Mossman N, Yosuf N, Marino M (2023). Implementation support for a social risk screening and referral process in community health centers. NEJM Catal Innov Care Deliv.

[12] Palakshappa D, Benefield AJ, Furgurson KF, Harley MG, Bundy R, Moses A, Taxter AJ, Bensinger AS, Cao X, Denizard-Thompson N, Rosenthal GE, Miller DP (2021). Feasibility of mobile technology to identify and address patients' unmet social needs in a primary care clinic. Popul Health Manag.

[13] Gottlieb LM, Hessler D, Long D, Laves E, Burns AR, Amaya A, Sweeney P, Schudel C, Adler NE (2016). Effects of social needs screening and in-person service navigation on child health: a randomized clinical trial. JAMA Pediatr.

[14] Steeves-Reece AL, Davis MM, Larson JH, Major-McDowall Z, King AE, Nicolaidis C, Goldberg B, Richardson DM, Lindner S (2023). Patients' willingness to accept social needs navigation after in-person versus remote screening. J Am Board Fam Med.

[15] Gurewich D, Linsky AM, Harvey KL, Li M, Griesemer I, MacLaren RZ, Ostrow R, Mohr D (2023). Relationship between unmet social needs and care access in a veteran cohort. J Gen Intern Med.

[16] Berkowitz SA, Seligman HK, Choudhry NK (2014). Treat or eat: food insecurity, cost-related medication underuse, and unmet needs. Am J Med.

[17] Teresi JA, Yu X, Stewart AL, Hays RD (2022). Guidelines for designing and evaluating feasibility pilot studies. Med Care.

[18] Ying X, Ehrhardt S (2023). Pilot trial characteristics, postpilot design modifications, and feasibility of full-scale trials. JAMA Netw Open.

[19] Weiner BJ, Lewis CC, Stanick C, Powell BJ, Dorsey CN, Clary AS, Boynton MH, Halko H (2017). Psychometric assessment of three newly developed implementation outcome measures. Implement Sci.

[20] (2019). RE-AIM.

[21] Glasgow RE, Harden SM, Gaglio B, Rabin B, Smith ML, Porter GC, Ory MG, Estabrooks PA (2019). RE-AIM planning and evaluation framework: adapting to new science and practice with a 20-year review. Front Public Health.

[22] Gaglio B, Shoup JA, Glasgow RE (2013). The RE-AIM framework: a systematic review of use over time. Am J Public Health.

[23] Brown EM, Loomba V, De Marchis E, Aceves B, Molina M, Gottlieb LM (2023). Patient and patient caregiver perspectives on social screening: a review of the literature. J Am Board Fam Med.

[24] De Marchis EH, Alderwick H, Gottlieb LM (2020). Do patients want help addressing social risks?. J Am Board Fam Med.

[25] Palakshappa D, Doupnik S, Vasan A, Khan S, Seifu L, Feudtner C, Fiks AG (2017). Suburban families' experience with food insecurity screening in primary care practices. Pediatrics.

[26] Palakshappa D, Goodpasture M, Albertini L, Brown CL, Montez K, Skelton JA (2020). Written versus verbal food insecurity screening in one primary care clinic. Acad Pediatr.

[27] Gottlieb L, Hessler D, Long D, Amaya A, Adler N (2014). A randomized trial on screening for social determinants of health: the iScreen study. Pediatrics.

[28] Cullen D, Woodford A, Fein J (2019). Food for thought: a randomized trial of food insecurity screening in the emergency department. Acad Pediatr.

[29] Miller DP, Foley KL, Bundy R, Dharod A, Wright E, Dignan M, Snavely AC (2022). Universal screening in primary care practices by self-administered tablet vs nursing staff. JAMA Netw Open.

[30] Chan DYL, Lee SWH, Teh PL (2023). Factors influencing technology use among low-income older adults: a systematic review. Heliyon.

[31] Straub ET (2017). Understanding technology adoption: theory and future directions for informal learning. Rev Educ Res.

[32] (2021). Mobile fact sheet. Pew Research Center.

[33] Agarwal AK, Ali ZS, Shofer F, Xiong R, Hemmons J, Spencer E, Abdel-Rahman D, Sennett B, Delgado MK (2022). Testing digital methods of patient-reported outcomes data collection: prospective cluster randomized trial to test SMS text messaging and mobile surveys. JMIR Form Res.

[34] Romeiser JL, Cavalcante J, Richman DC, Singh SM, Liang X, Pei A, Sharma S, Lazarus Z, Gan TJ, Bennett-Guerrero E (2021). Comparing email, SMS, and concurrent mixed modes approaches to capture quality of recovery in the perioperative period: retrospective longitudinal cohort study. JMIR Form Res.

[35] Garg A, Boynton-Jarrett R, Dworkin PH (2016). Avoiding the unintended consequences of screening for social determinants of health. JAMA.

