# Supplementary material for: Feasibility of Using Text Messaging to Identify and Assist Patients With Hypertension With Health-Related Social Needs: Cross-Sectional Study
Source: JMIR Cardio. 2024 Feb 13;8:e54530. doi: 10.2196/54530 (PMC10900090; doi:10.2196/54530)
Supplement: Multimedia Appendix 1 [file cardio_v8i1e54530_app1.docx]

**Initial Text Message**

From your care team at AHWFB: At Atrium Health Wake Forest Baptist, we want to provide the best care possible for our patients. To help us do that, we invite you to answer some questions about concerns patients may have that can affect their health. Reply STOP to opt-out of messages about this service.

[link to social risk questionnaire]

**Health-related Social Needs Text Message Questionnaire**

We are hearing from many of our patients that they are worried about different things at home, such as not having enough food to eat or having problems with transportation. These concerns can affect a person’s health, and we want to help. The purpose of this study is to test a new process to better connect our patients with services. You were randomly selected as one of 200 Atrium Health Wake Forest Baptist patients to complete this questionnaire. Depending on your answers, you may receive some information about services in the community. If you agree to a follow-up message, you may receive another message in about a month to see if the information was helpful.

Your participation in this study is voluntary. You do not have to participate in this study if you do not want to. You may or may not benefit from participation. Your responses to the questions will be entered into your medical record. If you do not want this information entered into your record, you do not have to complete this questionnaire.

1. What is your living situation today?

[ ] I have a steady place to live

[ ] I have a place to live today, but I am worried about losing it in the future

[ ] I do not have a steady place to live (I am temporarily staying with others, in a hotel, in a shelter, living outside on the street, on a beach, in a car, abandoned building, bus or train station, or in a park)

2. Think about the place you live. Do you have problems with any of the following? (check all that apply)

[ ] Pests such as bugs, ants, or mice

[ ] Mold

[ ] Lead paint or pipes

[ ] Lack of heat

[ ] Oven or stove not working

[ ] Smoke detectors missing or not working

[ ] Water leaks

[ ] None on the above

3. Within in the past 12 months, you worried that your food would run out before you got money to buy more.

[ ] Often true

[ ] Sometime true

[ ] Never true

4. Within in the past 12 months, the food you bought just didn’t last and you didn’t have money to get more.

[ ] Often true

[ ] Sometime true

[ ] Never true

5. Within the past 12 months, has lack of transportation kept you from medical appointments or from doing things needed for daily living?

[ ] Yes

[ ] No

6.   In the past 12 months has the electric, gas, oil or water company threatened to shut off services in your home?

[ ] Yes

[ ] No

[ ] Already shut off

If screens positive for one of the above, the following would display:

Would you like to receive information about resources in the community that may be available?

[ ] Yes

[ ] No

Would you allow us to follow up with you in about a month to see if this information was helpful?

[ ] Yes

[ ] No

**Subsequent text message 1**

If screens positive for any of the social risk questions and asks to receive information about resources, the following would display:

AHWFB: Thank you for completing the questions. You said you would like to receive information about services in the community. Please click the link below for a list. Reply STOP to opt-out of messages about this service.

[Link to list of services]

**Subsequent text message 2 (one month later)**

If screens positive for any of the social risk questions and asks to receive information about resources, the following would display:

AHWFB: About a month ago, you answered questions about some questions about resource needs. You should have received some information about services in your area. We are following up to see if this text process was helpful. Please click the link below to respond with your feedback. Reply STOP to opt-out of messages about this service.

[link to follow up questionnaire]

**Follow up questionnaire**

1. Since receiving the text message, did you use any of the resources that were provided? (Yes or No)

If yes,

- 1. Which ones?

1. The resources I received were helpful?
   1. Strongly agree
   2. Agree
   3. Neither agree or disagree
   4. Disagree
   5. Strongly disagree
2. I learned about community resources I had not known about before.
   1. Strongly agree
   2. Agree
   3. Neither agree or disagree
   4. Disagree
   5. Strongly disagree

|  | **Completely**  **disagree** | **Disagree** | **Neither agree nor disagree** | **Agree** | **Completely**  **agree** |
| --- | --- | --- | --- | --- | --- |
| 1. The text messaging process meets my approval. | 1 | 2 | 3 | 4 | 5 |
| 1. Receiving information via text messaging is appealing to me. | 1 | 2 | 3 | 4 | 5 |
| 1. I liked receiving the questions and the information by text message. | 1 | 2 | 3 | 4 | 5 |
| 1. I welcome being asked questions and receiving this information by text message. | 1 | 2 | 3 | 4 | 5 |
